# A novel nail-plate construct for the treatment of AO/OTA 31-A3.3 intertrochanteric fractures: a finite element analysis

**DOI:** 10.3389/fbioe.2025.1559765

**Published:** 2025-05-21

**Authors:** Jixing Fan, Yuan Cao, Zengzhen Cui, Shan Gao, Yang Lv, Fang Zhou

**Affiliations:** ^1^ Department of Orthopedics, Peking University Third Hospital, Beijing, China; ^2^ Engineering Research Center of Bone and Joint Precision Medicine, Ministry of Education, Beijing, China

**Keywords:** intertrochanteric fracture, AO/OTA 31-A3.3, PFUN, PFNA, finite element analysis

## Abstract

**Background:**

The AO/OTA 31-A3.3 is the most unstable type with a lesser trochanteric fragment and a broken lateral femoral wall (LFW), which constitute a four-part unstable intertrochanteric fracture. Implant failure remains one of the catastrophic consequences after surgical treatment. A novel nail-plate construct, called proximal femoral universal nail system (PFUN), is proposed by our team to fix comminuted LFW fracture fragment and lesser trochanteric fragment. The aim of this study is to evaluate the biomechanical properties of PFUN compared with proximal femoral nails anti-rotation (PFNA) for the treatment of AO/OTA 31-A3.3 intertrochanteric fractures.

**Methods:**

An AO/OTA 31-A3.3 intertrochanteric fracture model was established by computed tomography images. The models of implant (PFUN and PFNA) were created and virtually inserted into the A3.3 fracture model. The von Mises stress on the proximal femur, fracture end, implant and the total displacement of the device components were evaluated and compared for both PFUN and PFNA models.

**Results:**

The maximum von Mises stress in the proximal femur of the PFNA model increased by 85.81% when compared with the PFUN model in A 3.3 intertrochanteric fractures. The peak von Mises stress was located at the medial-inferior part of the fracture ends in the PFUN and PFNA models and the maximum von Mises stress in the PFUN model and PFNA model was 27.27 MPa and 49.95MPa, respectively. The PFUN model and PFNA model had similar peak von Mises stress in the implant. Furthermore, the maximum displacement in the PFUN model was much smaller than that in the PFNA model.

**Conclusion:**

The PFUN exhibited a lower peak von Mises stress in the proximal femur and fracture end, and a smaller maximum model displacement than PFNA in A3.3 intertrochanteric fractures. Our findings might provide valuable references for clinical decision making in surgical treatment of complex intertrochanteric fractures.

## Introduction

The intertrochanteric fracture is a common fragile fracture in the elderly, leading to the loss of independence and substantial economic burden. Early surgery is recommended, enabling early mobilization and avoiding bed-rest complications (Sing et al., 2023). For unstable intertrochanteric fractures, there is a tendency of hip varus and excessive collapse (Kregor et al., 2014). The AO/OTA 31-A3.3 intertrochanteric fracture is the most unstable type with a lesser trochanteric fragment and a broken lateral femoral wall (LFW), which constitute a four-part unstable intertrochanteric fracture. Despite great efforts made by orthopedic trauma surgeons, implant failure remains one of the serious complications after surgery for unstable intertrochanteric fractures (Hong et al., 2024; Fan et al., 2021). Therefore, it is very important to choosing a proper implant for unstable intertrochanteric fractures.

The lateral femoral wall, which was initially described by Gotfried, provided a lateral buttress for sliding of the head-neck fragment (Gotfried, 2004). In recent years, most researchers had realized that an intact LFW played an important role in the surgical stabilization of unstable intertrochanteric fractures (Palm et al., 2007; Fan et al., 2022). It had been reported that the LFW fracture was the primary independent predictor of fixation failure complication fixed with a compression hip screw (Palm et al., 2007). For these fractures, the intramedullary nail was the preferred choice for minimal surgical trauma, better biomechanical performance, and satisfactory functional outcomes. Although substantial evidence had proven that the nail itself could play the role of a lateral buttress and prevent excessive sliding of the head-neck fragment (Kim et al., 2015), intramedullary fixation encountered great difficulties in reducing or fixing the LFW when it was broken, which might increase the instability of unstable intertrochanteric fractures (Gao et al., 2018). Although a few methods had been introduced to reconstruct the lateral wall by adding an additional wire, screw or plate to nail, it could cause additional soft tissue dissection, bleeding, and increased surgical time (Wang et al., 2020; Jain et al., 2022). Furthermore, the additional fixation was not strong enough to maintain the postoperative stability, especially in osteoporosis or comminuted fractures, where there was a high risk of breakage, loosening and backout.

**FIGURE 1:**
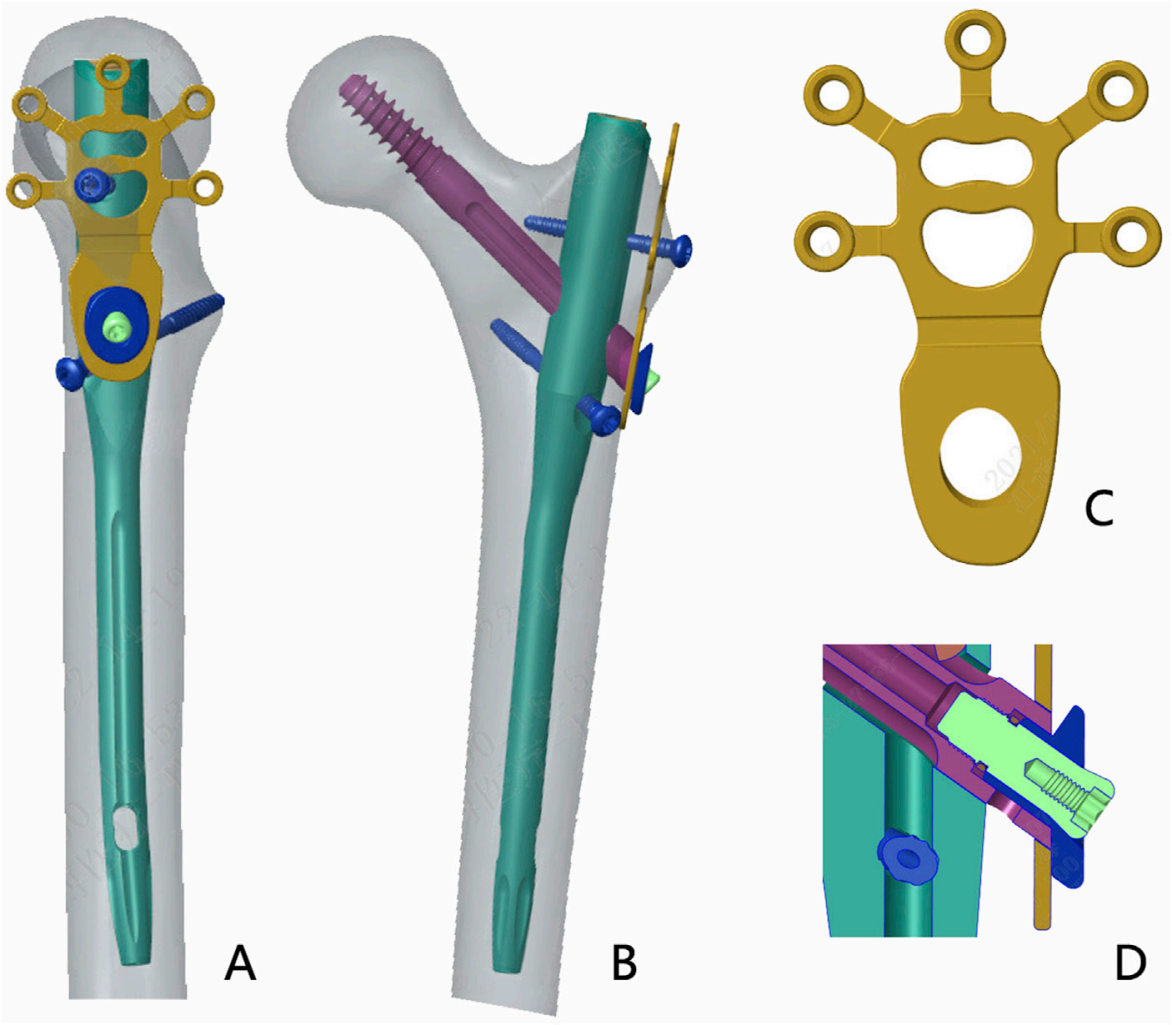
The schematic diagram of the proximal femoral universal system (PFUN) for A3.3 intertrochanteric fracture fixation. **(A)** The lateral view; **(B)** The front view; **(C)** The lateral wall plate; **(D)** The nail and lateral plate was hold together by one nut.

For reconstruction of the LFW integrity and improving postoperative stability, we designed a novel nail-plate construct called proximal femoral universal nail system (PFUN). This design had been patented in China (ZL2021111725422.3). In this internal fixation system, apart from the usual main nail, lag screw (or helical blade) and a lock nail, it consisted of a lesser trochanteric screw to fix the lesser trochanteric fragment and a lateral plate to fix the lateral femoral wall fragment. The lateral plate and the intramedullary nail were hold together with nuts. (Figure 1). This novel nail-plate construct was designed to fix comminuted LFW fractures. We assumed that this comprehensive internal fixation could have better biomechanical stability theoretically than commonly used proximal femoral nails anti-rotation (PFNA). The aim of this study was to evaluate the biomechanical properties of PFUN compared with PFNA for the treatment of AO/OTA 31-A3.3 intertrochanteric fractures.

## Materials and methods

### Finite element model establishment

**FIGURE 2:**
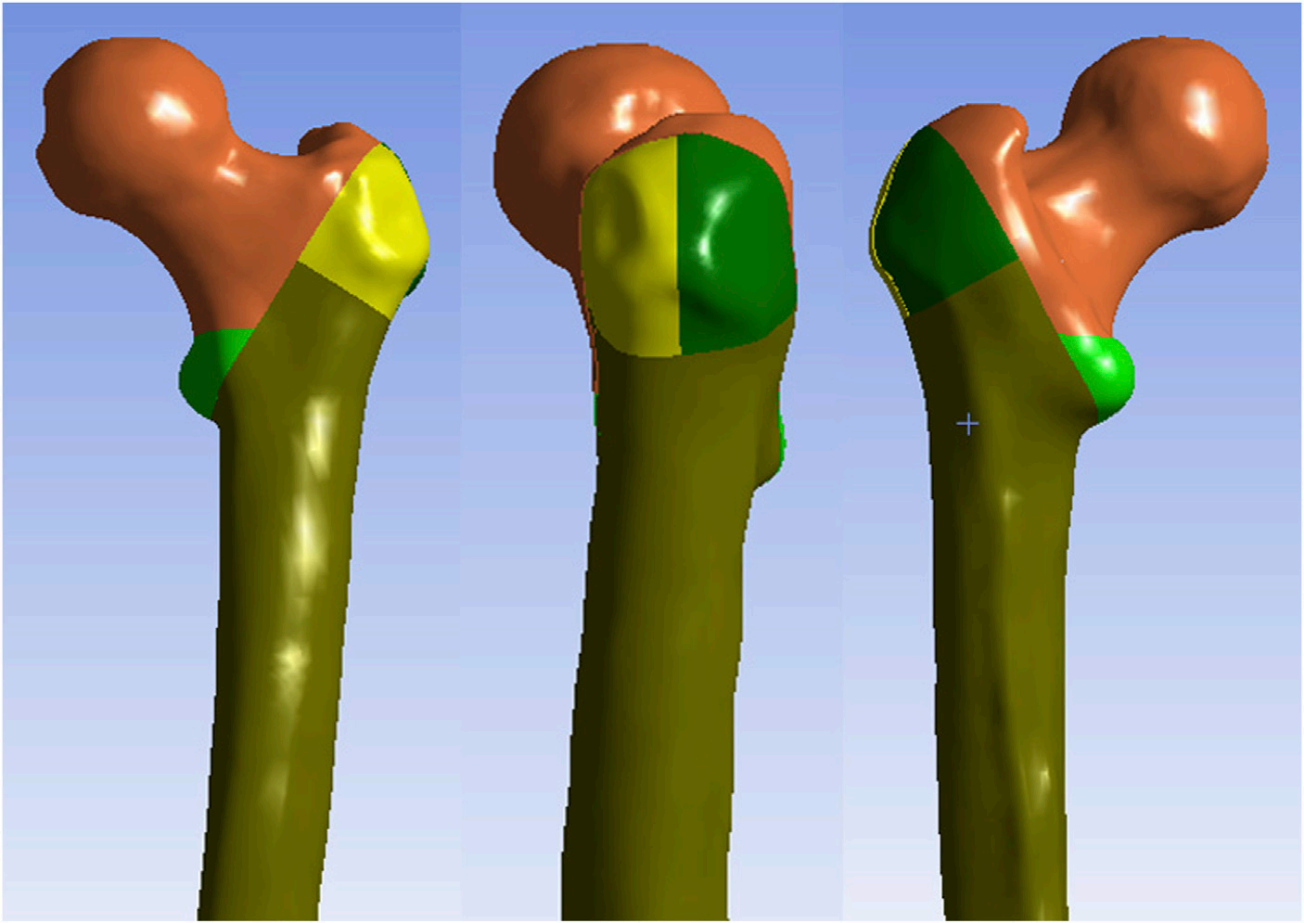
The AO/OTA 31A 3.3 intertrochanteric fracture.

In present study, one healthy Chinese male volunteer was recruited: age 65 years old, weight 70 kg, height 170 cm. The X-ray examination was performed, which showed that the femur was normal without any signs of femoral diseases or deformities. This 3D finite element model has been used in a previous study (Fan et al., 2022). The proximal femur was scanned with a 64-slice spiral CT (GE, USA), and the data were saved in Digital Imaging and Communications in Medicine (DICOM) format. Then, the femur data was imported into Mimics 17.0 software (Materialise, Belgium) and the three-dimensional (3D) model of the proximal femur was reconstructed from the CT images. The surface errors (spike, intersection, etc.) of the 3D model of the proximal femur were corrected in the Geomagic Studio 12.0 software (Raindrop Inc., USA). After the correction of the surface roughness of the model, the 3D smooth solid model was developed and imported into SolidWorks program (Dassault Systemes SolidWorks Corp., USA). Next, an intertrochanteric fracture with medial wall fracture and lateral wall fracture (AO/OTA 31A 3.3)) was created in SolidWorks 2017 software (Figure 2). The AO/OTA 31-A3.3 fracture was simulated according to the model described by Meinberg et al. (2018), with a major intertrochanteric fracture line, associated with a free lesser trochanteric fragment and a free bone fragment of the LFW.

**FIGURE 3:**
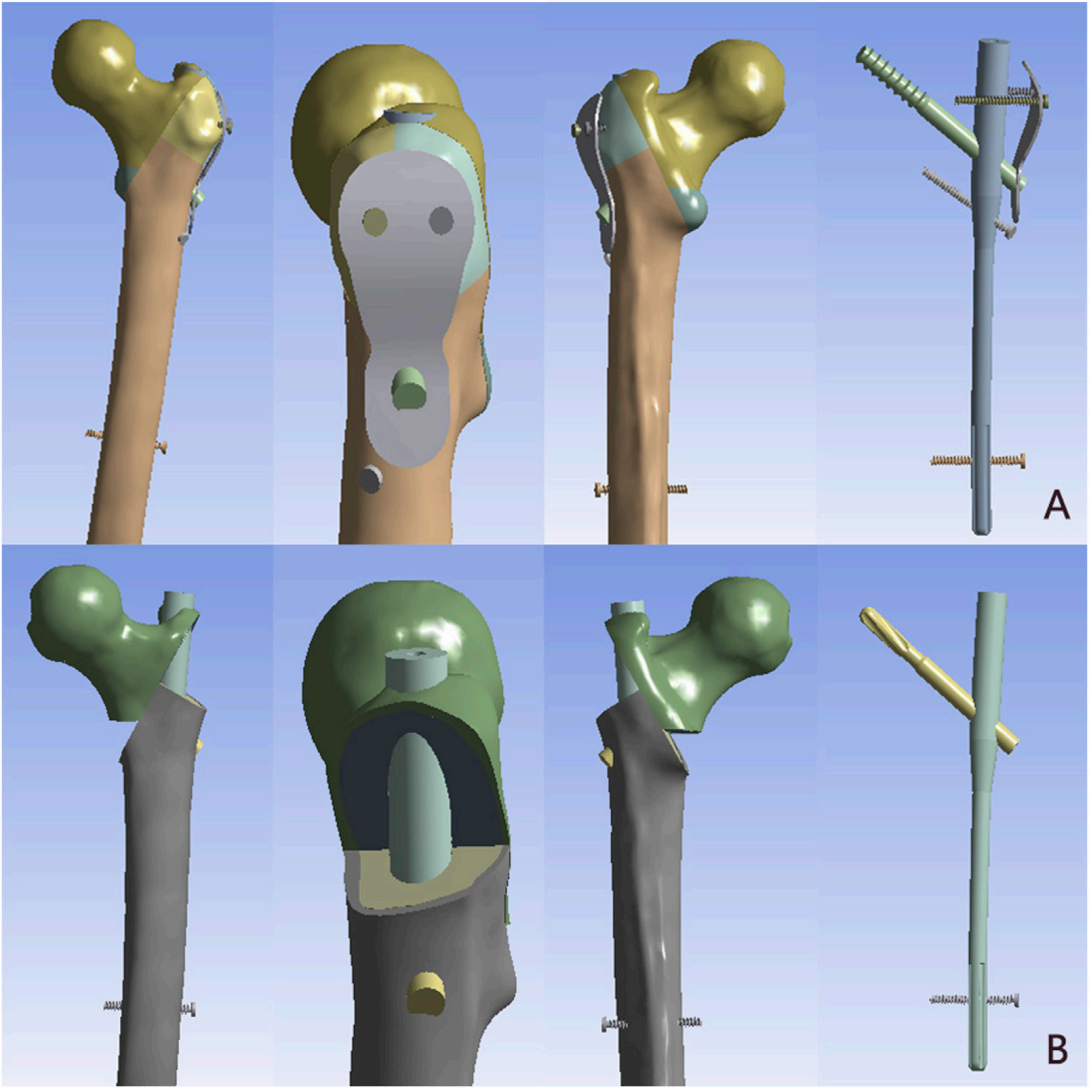
The model of AO/OTA 31A 3.3 intertrochanteric fracture were implanted with the PFUN and PFNA. **(A)** The PFUN; **(B)** The PFNA.

Then, the models of implant (PFUN and PFNA) were modeled by SolidWorks software according to the size of the intramedullary nail provided by the manufacturer. The implants were virtually inserted into the proximal femur. The spiral blade/lag screw were located in the middle and lower third of the femoral neck. The entry point was located at the apex of the greater trochanteric (Figure 3). Subsequently, the models were imported into ANSYS Workbench 14.5 (ANSYS Inc., Canonsburg, PA) for analysis.

The solid models were discretized into four-node tetrahedral elements using ANSYS Workbench. To evaluate the accuracy of finite element models, convergence tests were performed to determine the optimum maximum element size. After the convergence measurement, the mesh size was determined to be 2 mm.

In present study, all materials were assumed to be homogeneous, isotropic, and with linear elastic behavior (Henschel et al., 2016). The material properties of the femur and implant materials used in the models were summarized in Table 1 (Kwak et al., 2018). According to the well-established and approved test contact setup method described in previous studies, binding contact was formed between the internal fixation screw and the femur (Zhou et al., 2020). Friction contact was used on the fracture surface with a friction coefficient of 0.46.

**TABLE 1:** Material properties used in the simulations in this study.

Material	Young’s modulus (Mpa)	Poisson’s ratio
Cortical bone	17,000	0.33
Cancellous bone	1000	0.3
PFUN/PFNA (Ti-6Al-7NB)	110,000	0.35

### Boundary and loading conditions

For boundary condition, the distal end of the femur was constrained in all degrees of freedom. The loading forces acting on the femur presented the loads at the heel strike of normal walking (Lotz et al., 1995). A joint reaction force of 2,967.7 N ({x, y, z} = {1234.8, −352.8, - 2,675.4}) was applied at the femoral head (4.2 times body weight) (Lee et al., 2016).

### Observation index

In the finite element analysis, the peak von Mises stress on the proximal femur and implant, the total displacements of the models were selected as indices of the stability. They were evaluated and compared under the heel strike of normal walking.

## Results

### Von mises stress in the proximal femur

**FIGURE 4:**
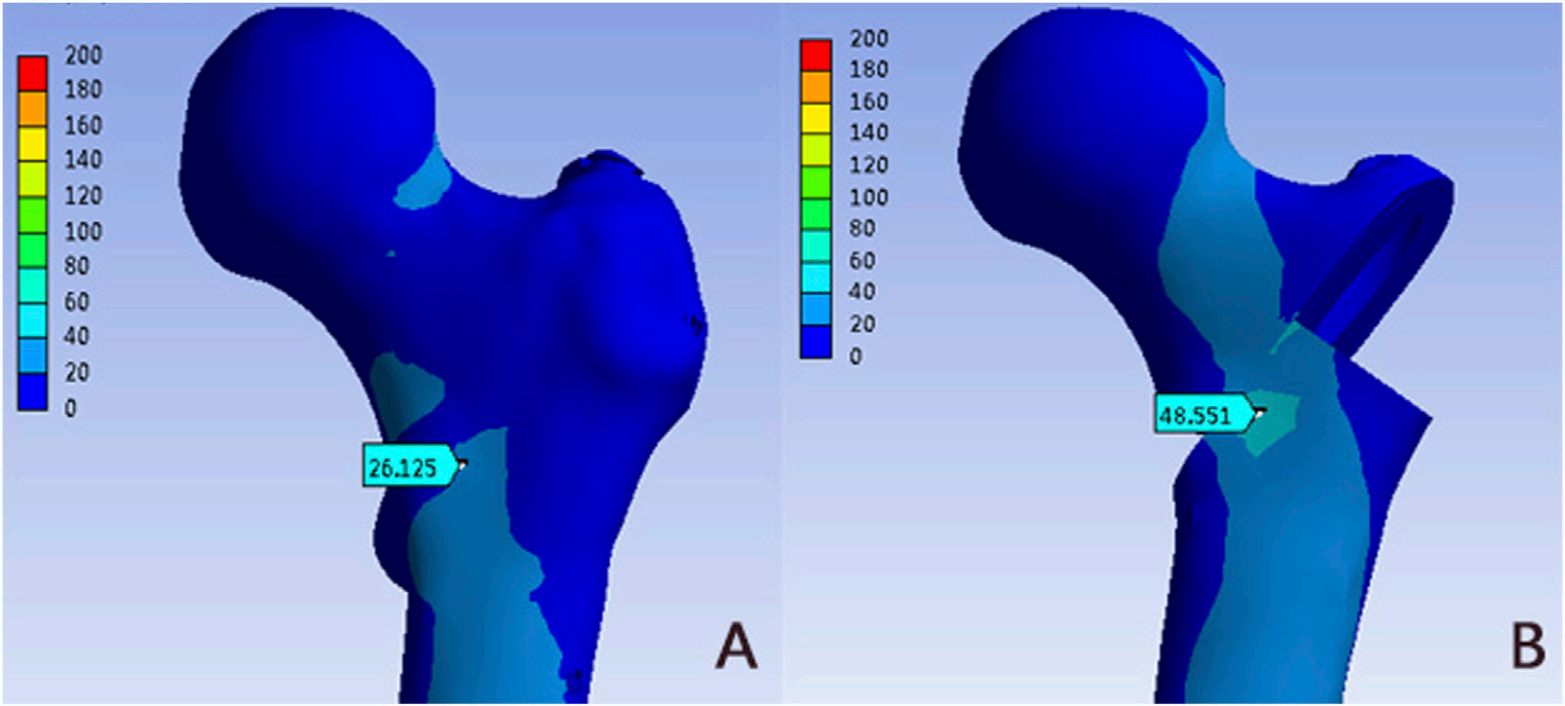
The Von Mises stress distribution (MPa) on the proximal femur: **(A)** PFUN model; **(B)** PFNA model.

The von Mises stress distribution of the proximal femur in PFUN and PFNA were shown in Figure 4 and Table 2. In two models, the stress concentration area was located at the medial-inferior part of the proximal femur; while differences in von Mises stress distribution were observed. The maximum von Mises stress of the PFNA model increased by 85.81% when compared with the PFUN model, and the magnitude of these two models were 26.13 MPa and 48.55 MPa, respectively.

**TABLE 2:** Parameters results.

Parameters	PFUN model	PFNA model
The maximum von Mises stress of the proximal femur (Mpa)	26.13	48.55
The maximum von Mises stress in the fracture ends (Mpa)	27.27	49.95
The maximum von Mises stress of the implant (Mpa)	389.26	392.13
The maximum displacement of the model (mm)	13.81	15.93

### Von mises stress distribution in the fracture ends

**FIGURE 5:**
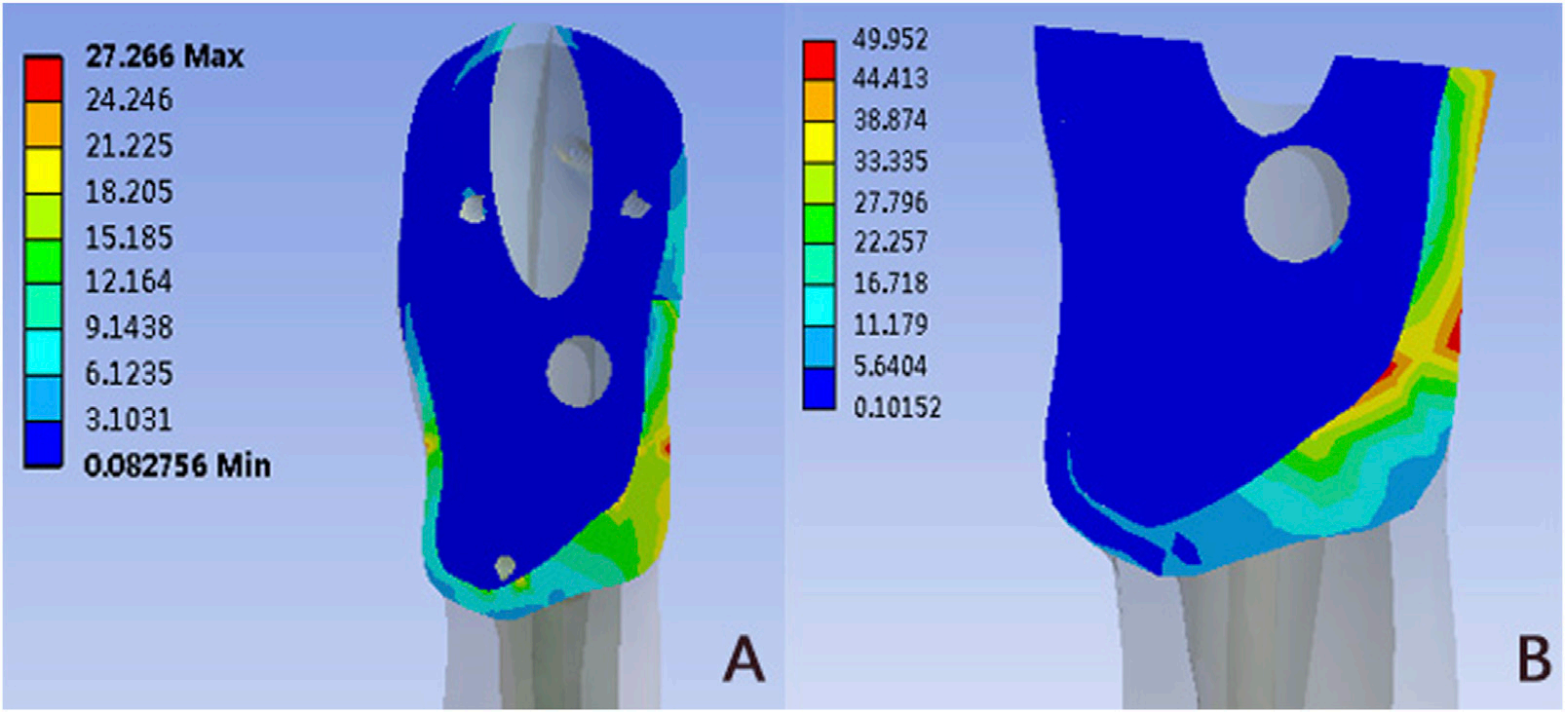
The Von Mises stress distribution (MPa) in the fracture ends: **(A)** PFUN model; **(B)** PFNA model.

Figure 5 and Table 2 showed the von Mises stress distribution in the fracture end for different models. The peak von Mises stress was located at the medial-inferior part of the fracture ends in the PFUN and PFNA models. The maximum von Mises stress in the PFUN model was 27.27MPa, and that of the PFNA model was 49.95MPa, respectively.

### Von mises stress distribution of implant

**FIGURE 6:**
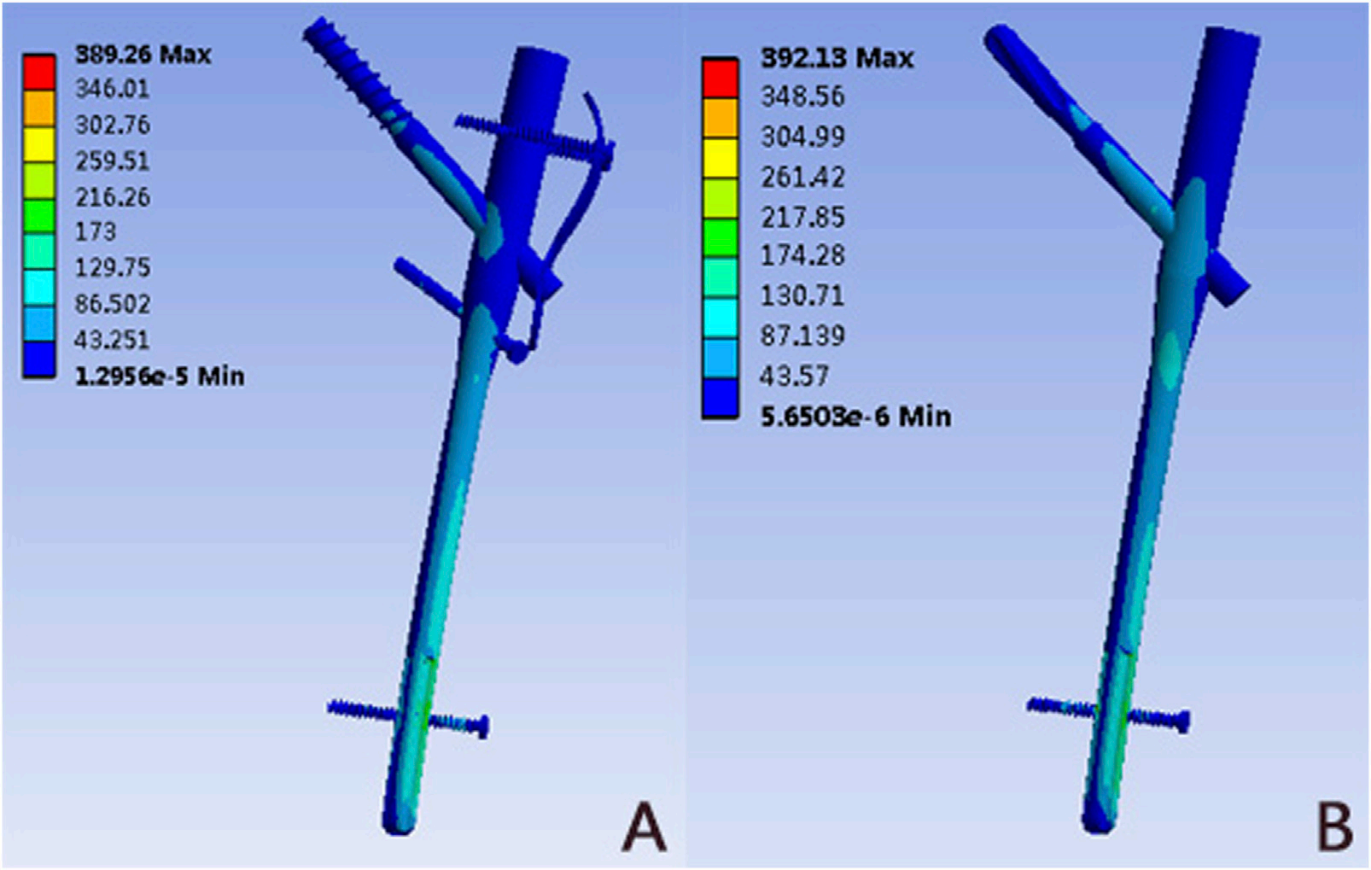
The Von Mises stress distribution (MPa) on the implant: **(A)** PFUN model; **(B)** PFNA model.

The von Mises stress distributions for two internal fixation models were assessed and shown in Figure 6 and Table 2. In these two implants, the stress was concentrated at the distal locking screw of each group. The PFUN model and PFNA model had similar peak von Mises stress, and the magnitude was 389.26 MPa and 392.13MPa, respectively.

### Model displacement

**FIGURE 7:**
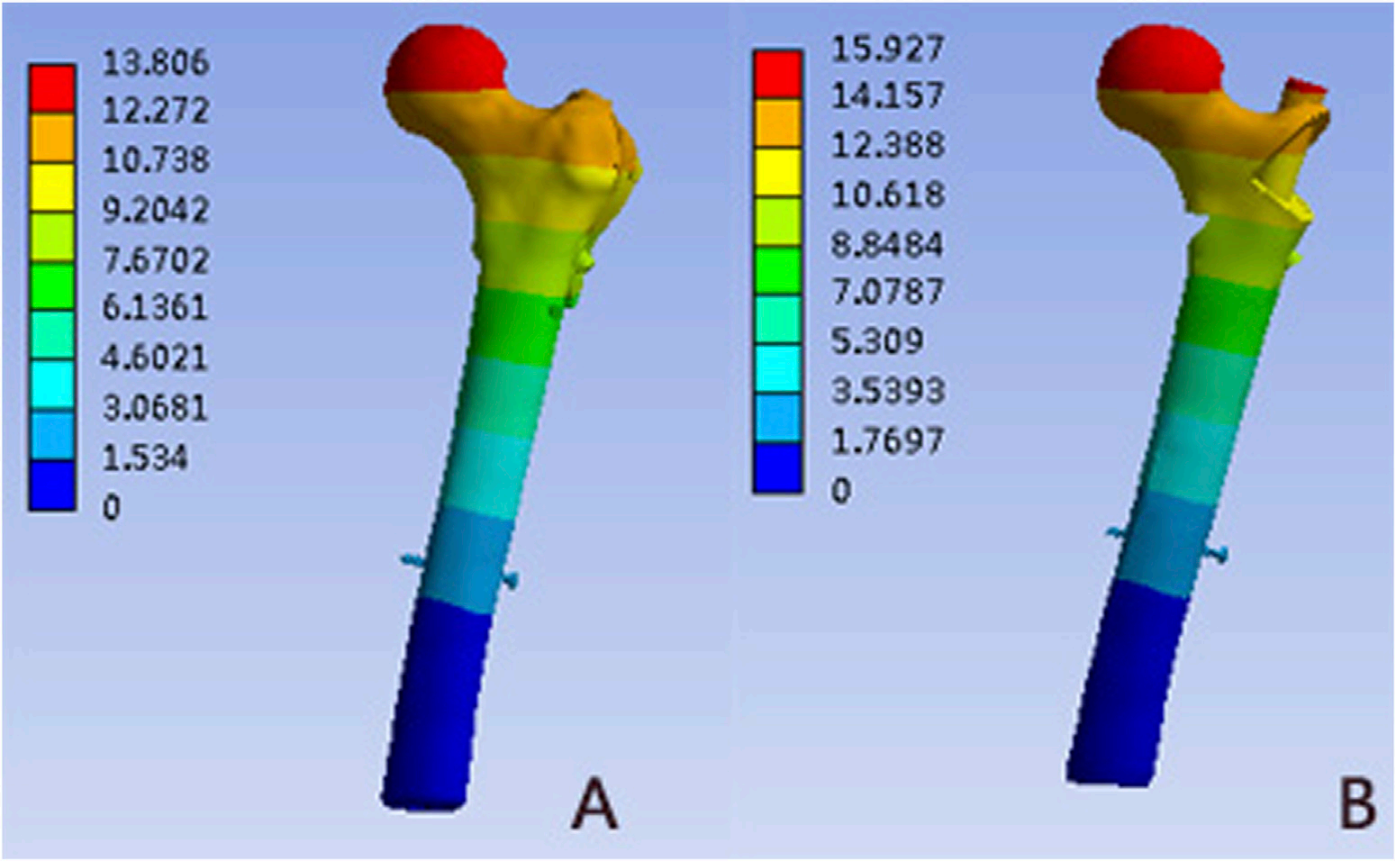
Displacement distribution (mm) in both models: **(A)** PFUN model; **(B)** PFNA model.

Figure 7 and Table 2 depicted the model displacement distribution in two models. The maximum displacements were located at the top of the femoral head for both models. The maximum von Mises stress of the PFNA model increased by 15.35% when compared with the PFUN model, and the magnitude of these two models were 15.93 mm and 13.81mm, respectively.

## Discussion

The AO/OTA 31-A3.3 intertrochanteric fracture is the most unstable type with a high risk of postoperative implant failure and secondary operation (Kregor et al., 2014). Currently, there are many difficulties in the treatment of the A3.3 intertrochanteric fracture for its complex fracture shape, poor arrangement of fragments and poor postoperative stability. Choosing an appropriate implant is a crucial step to successful treatment of these fractures. Conventional implants designed for intertrochanteric fractures struggled to accommodate the specific biomechanical demands of A3.3 fractures, leading to issues such as excessive medialization and potential distraction. In recent years, the use of intramedullary fixation was increasing because of its biomechanical superiority over extramedullary fixation. However, for these unstable intertrochanteric fracture with broken LFW, the nail alone had a tendency for varus collapse, screw back out, and implant failure (Shi et al., 2021). A few studies had reported that an additional plate was used to restore the lateral wall, so as to augment intramedullary nail (Wang et al., 2021; Zhao et al., 2024). However, the plate was applied separately without any fixation with the nail, i.e., free of hip or anti-rotation screws, with separate uni-cortical screws fixing the plate to the bone proximally and distally. In osteoporotic bones, the poor bone quality might cause plate loosening, fracture, or stress riser, as the plate was fixed with only uni-cortical screws both proximally and distally. Furthermore, it could cause additional soft tissue dissection, bleeding, and increased surgical time.

Therefore, we designed a novel intramedullary nail system called PFUN to hold the lateral plate and the nail together with one nut. Furthermore, one lesser trochanteric screw was applied to fix the lesser trochanteric fragment. In theory, this novel nail-plate construct had biomechanical superiority for the treatment of AO/OTA 31-A3.3 intertrochanteric fractures, which could fix the lesser trochanteric fragment and LFW fragment together tightly. In this finite element analysis, we constructed a model of an AO/OTA 31-A3.3 type intertrochanteric fracture, and compared the biomechanical properties of PFUN and PFNA in the treatment of A3.3 intertrochanteric fractures. From these results, PFUN presented a lower peak von Mises stress in the proximal femur and fracture end, and a smaller maximum model displacement, which suggested that PFUN had a biomechanical superiority in the treatment of A3.3 type intertrochanteric fractures.

The postoperative stability of intertrochanteric fracture relied on the intact medial wall to offer bone strut and LFW to provide lateral buttress. If the posteromedial section was unstable, the integrity of the lateral wall was decisive in maintaining the postoperative stability. The LFW fracture was serious enough to cause high degree of instability, leading to increased collapse, varus mal-reduction, shaft medialization, and increased re-operation rates (Mohamed et al., 2020). Although the intramedullary nail could provide lateral buttress, the nail alone was insufficient to compensate for the broken LFW for its disability to reduce and fix the LFW fragment. Various reconstruction methods of the broken LFW had been described while using screws, cerclage or plate (Zhao et al., 2024; Kulkarni et al., 2017). These were weak construct for resisting high forces around hip and fails to effectively buttress the lateral wall leading to breakage, back out, loosening, and mal-reduction, especially in osteoporotic bone. This novel nail-plate construct could hold the nail and plate together tightly to fix the comminuted LFW fragment closely. The additional plate could also reduce the interfragmentary shear movement and rotation. It was known that a reduction in interfragmentary shear micromotion was associated with faster fracture healing (Elkins et al., 2016). In this finite element analysis, we compared the biomechanical performance of the PFUN and PFNA and the results showed that the PFUN was superior to the PFNA biomechanically in the treatment of A3.3 intertrochanteric fractures. Therefore, this novel nail-plate construct might be an alternative for the reconstruction of the comminuted broken LFW.

**FIGURE 8:**
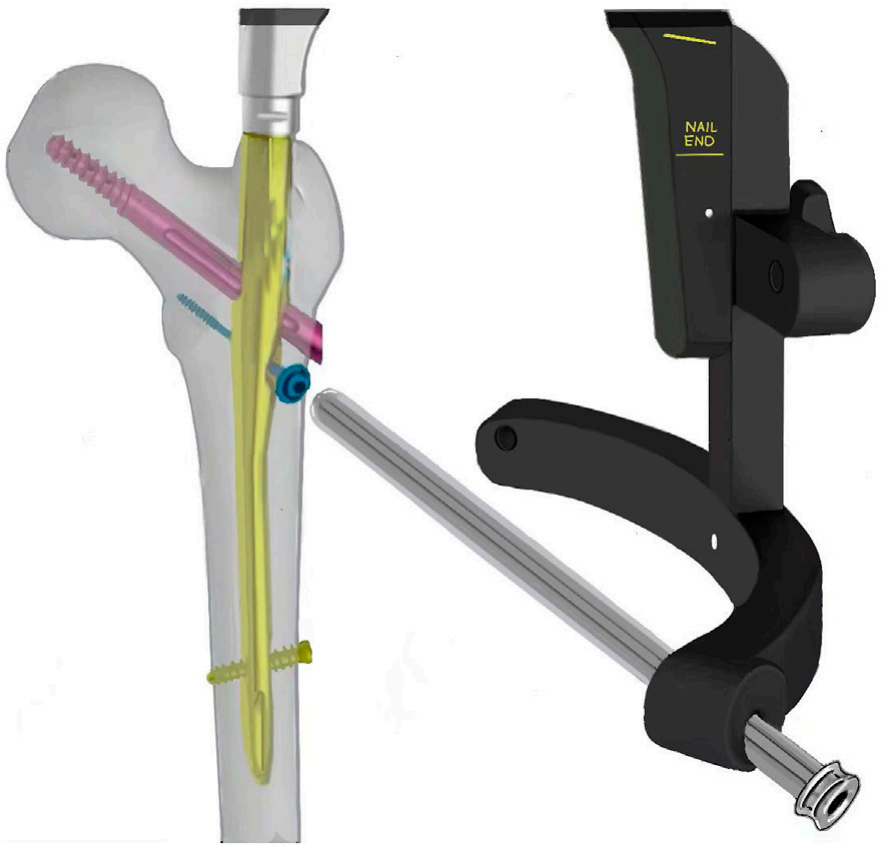
The illustration of a guide device for lesser trochanteric screw application.

The coxa varus might be caused by the loss of the medial support postoperatively due to the hinge (Nie et al., 2017). The medial wall consisted of the medial cortex and medial calcar located in its deep side. When a lateral wall fracture occurred, the medial support played an important role in maintaining the stability of the intertrochanteric fracture. One study reported that the presence of medial independent fragments was significantly correlated with the postoperative neck-shaft angle loss and femoral head collapse (Ren et al., 2020). Therefore, reconstructing the integrity of the medial wall was necessary. A cable was usually used to fix the lesser trochanter fragment (Kim et al., 2017). However, it was difficult to fix the lesser trochanteric fragment in clinical practice owing to technical restrictions, the longer operation time and greater blood loss. Therefore, we introduced a lesser trochanteric screw to fix the medial wall fragment by minimal minimally invasive surgical technique with a guide device. (Figure 8) In this study, we found that the stress concentration area was located at the medial-inferior part of the proximal femur in A3.3 intertrochanteric fractures and the intertrochanteric fracture treated with PFUN model showed a smaller stress concentration compared with the PFNA model. Similarly, the maximum stress in the fracture ends was also located at the medial-inferior part in both models, and the magnitude of PFNA model increased by 83.17% when comparing with PFUN model. This indicated that the application of the lesser trochanteric screw could decrease the stress concentration and a decrease in the peak von Mises stress of the proximal femur and fracture end could decrease the risk of coxa varus after daily loading. Furthermore, one study revealed that all patients in A1 group achieved bone union, while only 51.3% patients achieved bone union in A2 group after dynamic hip screw (DHS) implantation for intertrochanteric fractures (Hsu et al., 2013). Therefore, we thought that reducing and fixing the large lesser trochanteric fragment was useful and the PFUN could provide a relatively strong stability to prevent postoperative coxa varus for the treatment of A 3.3 intertrochanteric fractures.

For complex intertrochanteric fractures, postoperative biomechanical stability was closely related the good therapeutic effect. The main indicator for evaluating the stability of the A3.3 intertrochanteric fracture stabilized by a PFNA or PFUN device was the total displacement of the proximal femur and the internal fixation device after force. In this finite element analysis, the maximum displacement of the femur and implant in the PFUN group was 15.35% less than that of PFNA group, showing good postoperative stability. For A3.3 fractures, the absence of the medial and lateral support resulted in an imbalance of force loading on the femoral head, a varus or cutout complication might occur. For these complex intertrochanteric fractures, the implant was the only supportive mechanism capable of effective support. Furthermore, the A3.3 fractures could generate shear forces across the fracture site, resulting in medialization and shortening of the shaft with varus angulation and external rotation of the proximal fragment (Zhang et al., 2023). Therefore, it was vital to focus on minimizing movement between the fracture fragments to achieve relative stability during the early stage of fracture healing. In this study, the PFUN was capable of fixing the lesser trochanteric fragment and LFW fragment to minimizing fracture fragments movement, and a relatively smaller displacement was observed. Therefore, the PFUN might be an alternative for A 3.3 intertrochanteric fractures for its relatively better biomechanical stability.

This study had several limitations. First, the femur and implants were anisotropic materials. However, in this study, in order to reduce complexity of analysis, they were simplified into homogenous, isotropic and elastic materials. Although this study underwent some simplification and used conditions that might had differences with actual situations, it showed a clear trend for the topic being investigated. Second, the effect of soft tissues, such as the muscles and skin around the femur, on the forces on the femur after internal fixation was not considered in this study. Third, the obvious limitation of this study was that no experiments were carried out to verify the accuracy of the model. However, the purpose of this study is to compare relative values under the same loading environment and boundary conditions. As such, the lack of validation testing is justified. Moreover, the present study was based on the FEA of reconstruction models using CT images. However, the actual surgical procedure was more complicated compared with that of the experiment here. Therefore, this study was only a preliminary discussion, and further comparisons required a larger sample of clinical research applications.

## Conclusion

In summary, compared to the PFNA, the FEA results showed that the PFUN exhibited a lower peak von Mises stress in the proximal femur and fracture end, and a smaller maximum model displacement in AO/OTA 31-A3.3 intertrochanteric fractures. The reconstruction of the integrity of the LFW and medial wall could improve the load conduction and distribution and the nail-plate construct design could enhance the postoperative stability, potentially reducing the likelihood of implant-related issues. Therefore, the PFUN might be a new advanced alternative for the treatment of A3.3 intertrochanteric fractures and further clinical research was required to verify these results.

## Data Availability

The original data presented in the study are included in the article/supplementary material, further inquiries can be directed to the corresponding authors.

## Abbreviations

LFW, lateral femoral wall; PFUN, proximal femoral universal nail system; PFNA, proximal femoral nails anti-rotation; DICOM, Digital Imaging and Communications in Medicine; DHS, dynamic hip screw.
